# Immunological aspects of halo nevus (Sutton’s Nevus)

**DOI:** 10.3389/fimmu.2026.1729726

**Published:** 2026-02-04

**Authors:** Nika Hlača, Marijana Vičić, Larisa Prpić-Massari

**Affiliations:** Department of Dermatovenerology, Clinical Hospital Center Rijeka, Faculty of Medicine, University of Rijeka, Rijeka, Croatia

**Keywords:** *halo nevus* (Sutton’s nevus), CD8^+^ T cells, dendritic cells, immune checkpoints, melanocyte autoimmunity, melanoma regression, vitiligo

## Abstract

Halo nevus has historically been observed as a localized skin phenomenon surrounding a melanocytic nevus. However, emerging studies indicate that halo nevus serves as a model for immune-mediated, primarily CD8+ T cells–targeted destruction of melanocytes, similar to vitiligo or regressing melanoma. The aim of this mini-review is to summarize the current understanding of the immunological mechanisms underlying halo nevus formation, to highlight similarities and differences between halo nevus and vitiligo, as well as contrasts with regressing melanoma. Acknowledging halo nevus as a model for a controlled immune response against melanocytes could offer valuable insights into understanding triggers, potential antigens, and the balance of effector and regulatory responses, with translational relevance to pigment disorders and tumor immunology.

## 1 Introduction

**Figure 1:**
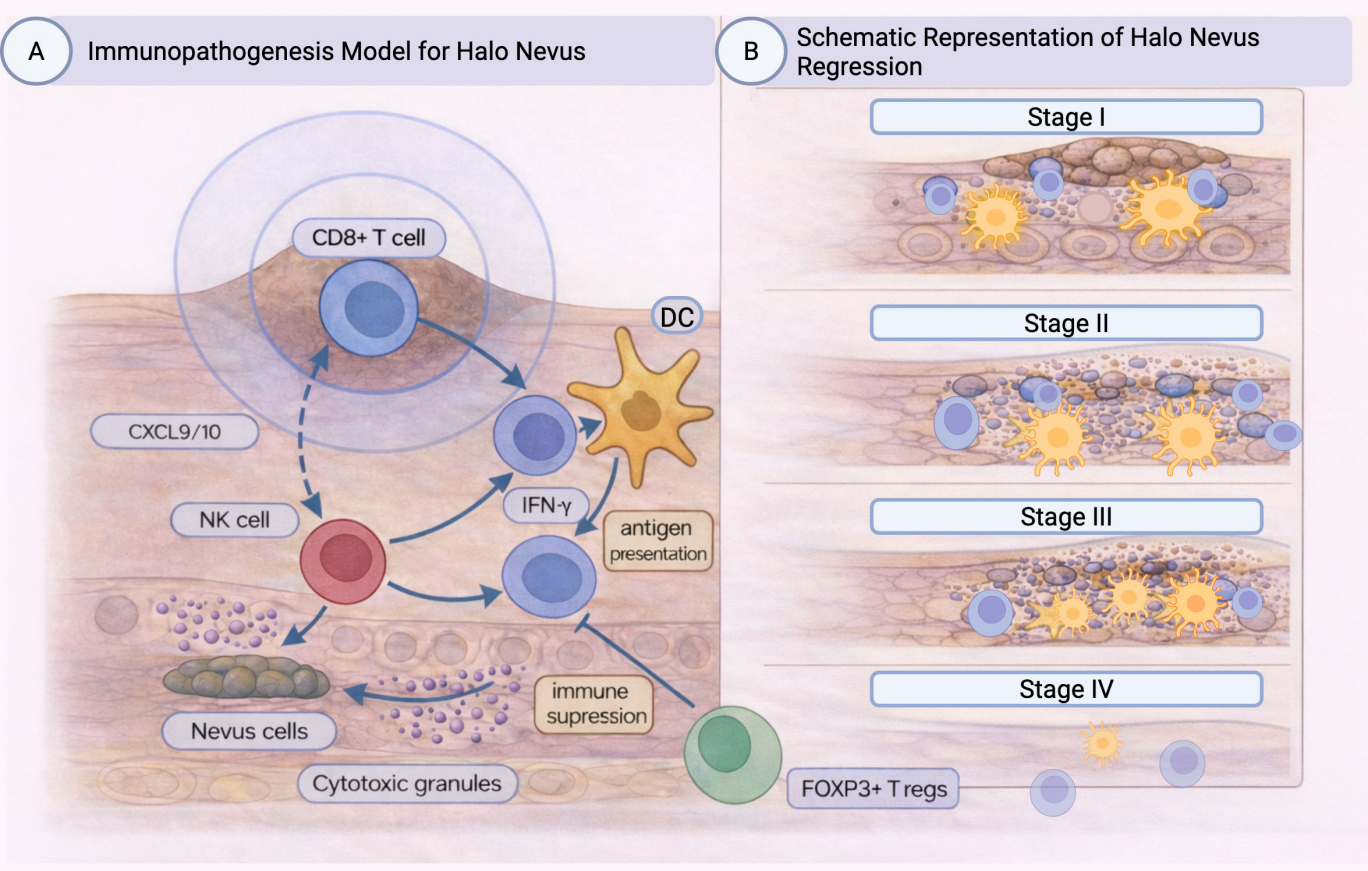
**(A)** Immunopathogenesis model for halo nevus (Sutton’s Nevus). Illustration of the immune mechanisms and pathways underlying halo nevus. DCs present melanocytic antigens to naive T cells, promoting their differentiation into CD8+ cytotoxic lymphocytes. The inflammatory response is driven primarily by IFN-γ and the chemokines CXCL9 and CXCL10, which recruit and activate effector CD8+ T cells and NK cells within the dermis. These cytotoxic cells release granzyme, granulysin, and perforin, leading to melanocyte apoptosis and formation of a perilesional depigmented halo. Simultaneously, regulatory mechanisms emerge, including PD-1/PD-L1 checkpoint signaling and FOXP3+ Tregs-mediated suppression, resulting in attenuation of cytotoxic activity and eventual repigmentation of the halo nevus. **(B)** Schematic representation of the histopathological stages of halo nevus regression. Stage I (pre-regression) shows an intact melanocytic nevus with early accumulation of Langerhans cells, in the absence of clinically visible depigmentation. Stage II (early regression) is characterized by blurred nevus nests, increasing infiltration of T lymphocytes and Langerhans cells, and initiation of melanocyte damage. Stage III (late regression) demonstrates sparse, poorly defined nevus cell remnants with dense, band-like inflammatory infiltrates composed predominantly of CD8^+^ T cells releasing cytotoxic granules, along with macrophages, and numerous antigen-presenting cells. Stage IV (complete regression) is defined by complete loss of nevus cells and marked reduction of the inflammatory infiltrate. Langerhans cells depicted in **(B)** represent epidermal dendritic cells and are conceptually aligned with DCs populations shown in **(A)**. Created in BioRender. Vicic, M. (2026) https://BioRender.com/2na2d3h.

A halo nevus (Sutton’s nevus) is a benign melanocytic lesion surrounded by a round rim of depigmentation caused by a local cytotoxic immune response against melanocytes (1). Halo nevi occur with equal frequency in both sexes and affect approximately 1% of the population (1). They most commonly appear in children and adolescents, mainly on the back, but also in the head and neck regions (1, 2). Approximately half of the patients have multiple halo nevi, which may be associated with vitiligo (3, 4). The association between halo nevi and vitiligo is not clear, although similar CD8+ cell-mediated immune mechanisms appear to be involved in both diseases (5, 6). Although the exact pathophysiological mechanism of halo nevus formation is unclear, halo nevi are thought to arise from a cell-mediated immune response directed against nevomelanocyte antigens (7). This immune response leads to regression and destruction of nevus cells, creating the characteristic ring of hypopigmentation in halo nevus lesions (**Figure 1A**). Halo nevi are therefore more common in patients with an increased number of nevi and/or a personal or family history of vitiligo (4, 8). Although uncommon, familial cases of halo nevi have also been reported (9). Halo nevi are less common in older age groups, hence regressing melanoma should be considered as a differential diagnosis in these cases (2, 10, 11). The halo phenomenon commonly occurs around acquired melanocytic nevi, but can also be seen in congenital melanocytic nevi, blue nevi, Spitz nevi, and melanoma (1, 2, 12).

### 1.1 Clinical and dermoscopic features of halo nevi

Halo nevi are melanocytic nevi surrounded by a round or oval halo of depigmentation measuring 0.5 to 5 cm (1, 2). The central melanocytic nevus is usually homogeneously pigmented, measures around 5 mm in diameter, with sharply demarcated, symmetrical borders (1). Over months or years, halo nevi undergo involution that ends with complete regression of the central nevus. This process begins with halo formation, followed by central pigment loss. Over time, the nevus completely disappears, leaving residual circular depigmentation, and in the final stage, spontaneous repigmentation occurs. These changes gradually occur over more than 10 years (1, 2, 13). Complete regression of the central nevus occurs in approximately 50% of cases (1, 2).

Currently, evidence on factors influencing halo diameter is limited. Rongioletti et al. found a significant correlation between these two parameters: the larger the nevus, the larger its halo. They proposed a “melanocyte antigenic unit” (MAU), a radial organization of melanocytes arranged around a central melanocytic lesion with which they share a phenotype and on which they are functionally dependent (14). In addition to halo nevi, the halo phenomenon can also occur around certain benign lesions such as seborrhoeic keratoses and dermatofibromas, as well as malignant tumors, including melanoma and basal cell carcinoma (12). In adults, new-onset halo lesions warrant careful evaluation, as the halo phenomenon can also occur around melanoma (15, 16). In addition, halo melanomas are extremely rare in children (3).

Dermoscopy is a useful non-invasive method for examining skin lesions with a dermoscope, which can assist in the evaluation of halo naevus (15). A globular pattern, a homogeneous pattern, or a combination of both are typical dermoscopic features of a halo naevus, while a reticular pattern is less common (15). The surrounding hypopigmented ring is usually pale, homogeneous, symmetrical, and sharply demarcated (10, 17).

## 2 Histopathological features of halo nevi

Four histological subtypes of halo nevus have been identified: (a) inflammatory, (b) non-inflammatory halo nevus, (c) halo nevus without a clinically visible halo, and (d) halo dermatitis surrounding a melanocytic nevus (2, 10). This classification is primarily based on the characteristics of the inflammatory infiltrate. Notably, this infiltrate may appear even before clinically evident depigmentation (10). Clinical correlation is essential, because the presence of inflammation does not always correspond with clinically visible changes. Some nevi may show a pronounced inflammatory infiltrate histologically without clinically visible halo, while non-inflammatory halo nevi may demonstrate a visible halo but lack inflammation under the microscope. Histologically, the central area of halo nevus typically shows a dense lymphocytic and histiocytic infiltrate. The presence and organization of nevus cells vary depending on the degree of regression. In fully regressed halo nevi, inflammatory cells may be present without any residual nevus cells (10). Halo nevi most commonly arise in the association with complex or intradermal nevi (1, 2). The inflammatory infiltrate differs in its density, cell composition, and pattern depending on the regression stage. In the initial phase (pre-regression), intact nevus with non-atypical nevus cells is present, accompanied by a moderate inflammatory infiltrate consisting of approximately 50% CD3+ T cells, 25% CD4+ T cells and a smaller population of Langerhans cells (LCs) and a dense, band-like inflammatory infiltrate composed of T lymphocytes surrounding centrally located nests of nevus cells (7, 18). In stage II (early regression), nevus nests show irregular borders and mild nuclear atypia. As regression begins, both T cells and LCs increase in number (2). The inflammatory infiltrate becomes more pronounced, surrounding and infiltrating the nests. S-100+ Langerhans cells are more prominent during early regression (2, 19, 20). In stage III (late regression), nevus cells appear as small, poorly defined clusters with nuclear atypia. The dense inflammatory infiltrate includes abundant factor XIIIa–positive (FXIIIa+) and S-100+ LCs (2, 19, 20). Moreover, during the late phase of regression, a dense infiltrate of T lymphocytes, mainly CD8+ along with some CD4+ cells can be seen between and within the nests of nevus cells. Most of these T cells express granzyme B, perforin, and Fas ligand (FasL), indicating their active cytotoxic function (2, 11, 18). Scattered CD68+ macrophages are also observed within the lesion (11, 19). In stage IV (complete regression), nevus cells are no longer present and the inflammatory infiltrate is markedly reduced or absent (2, 19) (**Figure 1B**). Studies have demonstrated that halo nevi can display a broad spectrum of cytological atypia, ranging from mild to severe. As a result, halo nevi should not be regarded as a single uniform clinicopathological entity, but rather as a diverse group with variable features (10).

## 3 Immunopathogenesis of halo nevus

Recent research suggests a key role for the cytotoxic T cell response in the pathogenesis of halo nevus (5) (**Figure 1A**). CD8+ T lymphocytes are present during all phases of halo nevus development, and they become predominant with progression of regression, reaching the highest proportions in late-stage lesions (2, 11). Most T lymphocytes in halo nevus lesions express granzyme B, perforin, FasL, and granulysin (GNLY), indicating strong cytotoxic activity (5, 7, 18, 21). Apart from CD8+ T lymphocytes, recent transcriptomic analysis of the inflammatory infiltrate in halo nevus found predominantly dendritic cells (DCs) and macrophages, while levels of natural killer (NK) cells, regulatory T cells (Tregs), and T helper 2 (Th2) cells were decreased (22). Transcriptomic analyses of halo nevus lesions suggest activation of regulated cell-death programs, including inflammatory cell death pathways (e.g., pyroptosis) and lipid peroxidation–driven death (ferroptosis), alongside apoptosis (22). In halo nevus, both human leukocyte antigen class I (HLA-I) and class II (HLA-II)-associated genes are upregulated, with an HLA-I predominance (5). Transcriptomic analyses have demonstrated that differentially expressed genes in halo nevus lesions are enriched in immune and interferon-related pathways, including responses to infectious stimuli, which closely overlap with signatures observed in vitiligo (23). These findings suggest that environmental factors, such as infections, may contribute to immune activation in susceptible individuals; however, the specific antigenic targets driving HLA-I–restricted cytotoxic responses in halo nevus remain unknown (23).

### 3.1 The role of innate immunity

DCs are professional antigen-presenting cells that play a key role in the initial recognition of foreign or self-antigens, and in the subsequent priming of T cells. In vitiligo, melanocyte oxidative stress activates DCs. Interestingly, similar oxidative stress reaction has recently been observed in halo nevus. The critical metabolite associated with the oxidative stress response in halo nevi lesions is uridine diphosphate–glucose (UDP-G), which may activate the innate immune system, particularly DCs. UDP-G belongs to a class of molecules known as danger-associated molecular patterns (DAMPs), which are released in response to cellular damage or oxidative stress (13, 24). Recently, Jiang et al. found upregulation of UDP-G which correlated with infiltration of immune cells such as CD8+ T cells and DCs (22). Transcriptomic analyses indicate that dendritic cell infiltration in halo nevus lesions is strongly associated with activation of multiple regulated cell-death pathways, including pyroptosis and ferroptosis, alongside apoptosis and disulfidptosis (22). Apart from DCs, neutrophils may also contribute to melanocyte death in halo nevus. Namely, neutrophils may induce NETotic cell death, a relatively rare form of cell death involving the release of neutrophil extracellular traps (NETs) (25). In halo nevi, programmed death ligand 1 (PD-L1)-expressing neutrophils are found in vicinity of CD8+ T cells, where they act immunosuppressively by inhibiting CD8+ T cells and thus leading to gradual repigmentation of halo nevi (26). Other immune cells in the infiltrate include macrophages; however, their functional polarization in halo nevus remains incompletely characterized, and transcriptomic findings showing reduced Th2-associated pathways and regulatory T cells suggest a context-dependent macrophage state (6).

In addition, recent transcriptomic analysis of the inflammatory infiltrate in halo nevus has revealed a predominance of DCs, T cells and macrophages, while NK cells, Tregs, and Th2 cells were comparatively reduced (22). NK cells may contribute to early stages of halo nevus development through direct cytotoxicity and immunomodulatory cytokine production (27). They possess a cytotoxic effect, but also enhance the immune response with numerous chemokines and cytokines, leading to accumulation of other immune cells (27). Recently, NK cells expressing granulysin (GNLY) have been demonstrated in lesions of halo nevus, infiltrating the nevus cells. However, in comparison to CD8+ T cells expressing GNLY, fewer CD56+ NK cells expressing GNLY were found in lesions of halo nevi (21).

### 3.2 Adaptive immune response

CD8+ T cells are critical for the destruction of melanocytes in halo nevi. Upon activation, CD8+ T cells express various cytotoxic molecules, such as perforin, granzyme, and FasL. Recently, granulysin (GNLY), an additional cytotoxic mediator, was identified in CD8+ T cells surrounding melanocytes in halo nevus lesions (21). Notably, compared to vitiligo, the expression of GNLY was much higher in halo nevus lesions than in vitiligo, which may indicate a stronger cytotoxic immune response in halo nevus (21). GNLY has cytolytic, antimicrobial, proinflammatory, chemoattractant, and tumoricidal functions. It has also been shown to act as an immune alarmin that stimulates the immune response and induces the recruitment and activation of antigen-presenting cells, such as DCs, as well as CD4+ and CD8+ αβ T cells, monocytes, and NK cells (28). In addition to CD8+ T cells, recent studies have found CD4+ cells and forkhead box P3–positive regulatory T cells (FOXP3+ Tregs) in halo nevus (11, 29). There was no difference in the expression of these cells in repigmented versus non-repigmented halo nevus. However, in cases where the depigmented halo persisted, CD8+ cells were still present. In contrast, in improved cases, where repigmentation of the halo occurred spontaneously, there was an interaction between CD8+ cells and CD68+ macrophages and CD20+ B cells (30).

#### 3.2.1 Immune checkpoints and regulatory pathways

Recently, programmed death ligand 1 (PD-L1) expression levels have been compared between halo nevi with spontaneous repigmentation and halo nevi without repigmentation. The group with repigmentation following halo nevus had significantly lower expression of PD-L1 (30). In cases where repigmentation occurred spontaneously, CD21-positive DCs accumulated around PD-L1+ cells (30). Mechanistically, activated CD8^+^ T cells produce interferon-γ (IFN-γ), a key cytokine that induces PD-L1 expression on multiple immune and stromal cell types, including neutrophils and macrophages (31, 32). This IFN-γ–PD-L1 axis functions as a negative feedback loop that limits excessive cytotoxic T-cell activity within the local tissue microenvironment.

## 4 Association between halo nevus and vitiligo

Halo nevi may be associated with autoimmune diseases, especially vitiligo, with which they share immunopathogenesis (4, 6, 8). Halo nevi are often observed in children with pre-existing vitiligo, and they may appear earlier in life in patients with vitiligo (33). However, it is not yet clear whether halo nevi are a risk factor for the development of vitiligo (8). A higher frequency of halo nevi has been observed in children with generalized vitiligo compared to those with localized forms of the disease (3, 33). Although several studies have associated the occurrence of multiple halo nevi with vitiligo, van Geel et al. believe that the risk of vitiligo decreases when the number of halo nevi exceeds three (8). Zhou et al. have shown that patients with halo nevi and the Koebner phenomenon have a higher risk of developing vitiligo (34). Additionally, patients with halo nevi have an increased risk of developing vitiligo if a family member has vitiligo (8). Halo nevus and vitiligo are both immune-mediated disorders characterized by well-defined depigmented skin lesions (35). In both conditions, cytotoxic CD8+ T cells target melanocyte antigens within the epidermis or melanocytic nevi, resulting in pigment loss. Although vitiligo and halo nevus share clinical, histopathological, and immunological similarities, their precise connection remains unclear (5, 33). Transcriptomic and gene expression analyses reveal overlapping molecular profiles between vitiligo and halo nevus, including interferon-driven and cytotoxic T-cell–associated signatures as well as antigen processing pathways, supporting their shared immunopathogenesis (23). Furthermore, genome-wide association studies in vitiligo have identified genetic loci shared with many autoimmune diseases, such as Hashimoto disease, alopecia areata, and pernicious anemia (35). Recent evidence suggests that patients with halo nevus may have a similar predisposition to systemic autoimmune diseases, reinforcing the view that both entities belong to a common autoimmune spectrum (6). Accordingly, halo nevus has been proposed as a localized model for studying vitiligo, due to the similar local immune response mediated by CD8+ T cells and the key cytokine mediator IFN-γ (5).

## 5 Halo nevus and melanoma immunology

Melanoma, a malignant tumor of melanocytes, can undergo partial or complete regression, a phenomenon that clinically resembles halo nevi. Regression results from a complex interaction between the host immune response and tumor antigens, with selective destruction of tumor melanocytes by cytotoxic lymphocytes. Like halo nevi, regressing melanomas contain dense lymphocytic infiltrates composed mainly of CD8+ T cells and macrophages (11, 36, 37). Both lesions may share overlapping histological features, particularly in the early phases of regression, which can complicate diagnosis. Nonetheless, several distinguishing characteristics can help in their differentiation. In halo nevi, proliferating melanocytes typically demonstrate maturation with depth, with atypical cells confined mainly to the superficial nests, while the deeper portion of the lesion contains smaller, more mature-appearing melanocytes. In contrast, melanoma characteristically shows a lack of maturation, with cytological atypia extending throughout the lesion and an overall disorganized architectural pattern. The nature and distribution of the inflammatory infiltrate can also provide diagnostic clues. Although early melanoma regression may exhibit an inflammatory response similar to that of halo nevi, the pattern differs: in halo nevi, the infiltrate is symmetrical and band-like, encircling the entire lesion, while in melanoma, it is typically asymmetrical, surrounding only selected tumor areas (10, 11). However, Brugués et al. report similar pattern of fibrosis in both entities (38). Immunohistochemical profiling further refines the distinction between these entities. Early-stage halo nevi demonstrate an increased number of FOXP3+ regulatory T cells, whereas regressing melanomas generally contain fewer such cells (11, 38). The CD4:CD8 T cell ratio also distinguishes halo nevi from melanomas. Halo nevi typically exhibit a CD8+ predominant infiltrate, while melanoma regression tends to show a CD4+ predominant infiltrate (11). Moreover, halo nevi display a more robust cytotoxic immune response, reflected by higher granzyme expression, and exhibit increased programmed cell death protein 1 (PD-1) expression, consistent with sustained T-cell activation accompanied by inhibitory regulatory feedback compared with melanoma (10, 11). Human melanoma black 45 (HMB-45) staining can also aid in differentiating between halo nevus and regressing melanoma, although it is not definitive on its own. In halo nevi, melanocytes within the superficial dermis are typically HMB-45-positive, while those in the deeper dermis lose expression, reflecting normal maturation. Conversely, melanoma often demonstrates irregular or diffuse HMB-45 positivity throughout the lesion, consistent with the lack of maturation. However, it is important to note that atypical HMB-45 expression patterns have occasionally been observed in halo nevi, which limits the marker’s diagnostic specificity and reliability (11, 36, 37). Despite these similarities, comparative immunophenotypic analyses reveal significant differences between these two entities. In comparison to regressing melanomas, halo nevi have a higher CD8/CD3 ratio, as well as increased expression of immune-regulatory markers such as PD-1, FOXP3, and CD25 (11, 38). Together, these data support a balanced cytotoxic–regulatory immune response in halo nevi, whereas regressing melanoma exhibits a distinct immune profile with reduced compensatory immune regulatory mechanisms (11, 29, 31, 38). The summary of key immunologic differences between halo nevus, vitiligo, and regressing melanoma is presented in **Table 1**.

**Table 1:** Key immunologic differences between halo nevus, vitiligo, and regressing melanoma.

	Halo nevus	Vitiligo	Regressing melanoma
Immunologic significance	Local model of immune-mediated melanocyte destruction with regulatory balance	Systemic immune-mediated disease targeting melanocytes	Tumor regression driven by host immune response
Main effector cells	CD8+ T cells >>CD4+ T cells; DCs; macrophages; NK cells; neutrophils	CD8+ T cells/Th1; DCs; NK cells	CD8+ and CD4+ T cells (often CD4-predominant); macrophages
Inflammatory pattern	Dense, band-like, symmetrical infiltrate	Subepidermal lesional and perilesional infiltrate	Patchy, asymmetrical infiltrate with fibrosis
Cytotoxic mediators	granzyme B, perforin, granulysin, FasL	granzyme B, perforin, granulysin, FasL	Variable; less intense than in halo nevus
Cytokine milieu	IFN-γ-driven inflammatory response	Predominantly IFN-γ–driven Th1 inflammatory response within a complex cytokine network	Mixed; immunosuppressive cytokines (TGF-β, IL-10)
Regulatory/Checkpoint profile	FOXP3+ Tregs and PD-1/PD-L1 upregulated	Reduced Tregs; PD-L1 variably expressed	Fewer FOXP3+ Tregs than halo nevus; variable PD-L1 expression
Outcome	Nevus regression ± repigmentation	Chronic depigmentation ± repigmentation	Partial/complete regression of melanoma, but malignancy persists

DCs, dendritic cells; FasL, Fas ligand; FOXP3, forkhead box P3; IFN-γ, interferon-gamma; IL-10, interleukin-10; NK, natural killer; PD-1, programmed cell death protein 1; PD-L1, programmed death ligand-1; TGF-β, transforming growth factor beta; Th1, T-helper 1; Tregs, regulatory T cells.

## 6 Conclusion

A halo nevus is a benign melanocytic lesion surrounded by a zone of depigmentation caused by a local immune reaction against melanocytes. The primary mediators of melanocyte death are cytotoxic T cells, supported by innate cells including NK cells, DCs, and macrophages, which contribute to the early stages of halo nevus development. Cytotoxic CD8+ T cells express various cytotoxic molecules that mediate melanocyte destruction. Understanding the immune mechanisms involved provides insights into tolerance breakdown and checkpoint regulation, which may be shared with vitiligo and melanoma. Therefore, halo nevus could serve as a model for studying immune-mediated reactions against melanocytes, particularly vitiligo and regressing melanoma.
