# Supplementary material for: Immunological aspects of halo nevus (Sutton’s Nevus)
Source: Front Immunol. 2026 Feb 4;17:1729726. doi: 10.3389/fimmu.2026.1729726 (PMC12913448; doi:10.3389/fimmu.2026.1729726)
Supplement: Supplementary file 1 [file DataSheet1.docx]

# AI Disclosure Prompts

Model and Source:
ChatGPT (GPT-5, OpenAI, 2025)

---

## Initial Prompt

Please review and improve the academic writing, coherence, and clarity of the following section from a biomedical review. Maintain all factual content and scientific terminology.

## Final Prompt

Proofread the full manuscript for grammar, punctuation, and journal-style consistency. Retain all original meaning and citations.

---

## Scope of AI Use

The AI tool was used solely for language editing and stylistic improvement. All factual content, references, and scientific interpretations were created, verified, and approved by the authors.
